# Insights from the Twittersphere: a cross-sectional study of public perceptions, usage patterns, and geographical differences of tweets discussing cocaine

**DOI:** 10.3389/fpsyt.2024.1282026

**Published:** 2024-03-19

**Authors:** Consuelo Castillo-Toledo, Oscar Fraile-Martínez, Carolina Donat-Vargas, F. J. Lara-Abelenda, Miguel Angel Ortega, Cielo Garcia-Montero, Fernando Mora, Melchor Alvarez-Mon, Javier Quintero, Miguel Angel Alvarez-Mon

**Affiliations:** ^1^ Department of Psychiatry and Mental Health, Hospital Universitario Infanta Leonor, Madrid, Spain; ^2^ Department of Medicine and Medical Specialities, Faculty of Medicine and Health Sciences, University of Alcala, Alcala de Henares, Spain; ^3^ Ramón y Cajal Institute of Sanitary Research (IRYCIS), Madrid, Spain; ^4^ Cardiovascular and Nutritional Epidemiology, Institute of Environmental Medicine, Karolinska Institute, Stockholm, Sweden; ^5^ IMDEA-Food Institute, Universidad Autónoma de Madrid, Consejo Superior de Investigaciones Científicas, Madrid, Spain; ^6^ Departamento Teoria de la Señal y Comunicaciones y Sistemas Telemáticos y Computación, Escuela Tecnica Superior de Ingenieria de Telecomunicación, Universidad Rey Juan Carlos, Fuenlabrada, Spain; ^7^ Department of Legal Medicine and Psychiatry, Complutense University, Madrid, Spain; ^8^ Service of Internal Medicine and Immune System Diseases-Rheumatology, University Hospital Príncipe de Asturias, (CIBEREHD), Alcalá de Henares, Spain

**Keywords:** cocaine, Twitter, social perception, infodemiology, drug use/abuse, geolocalization

## Abstract

**Introduction:**

Cocaine abuse represents a major public health concern. The social perception of cocaine has been changing over the decades, a phenomenon closely tied to its patterns of use and abuse. Twitter is a valuable tool to understand the status of drug use and abuse globally. However, no specific studies discussing cocaine have been conducted on this platform.

**Methods:**

111,508 English and Spanish tweets containing “cocaine” from 2018 to 2022 were analyzed. 550 were manually studied, and the largest subset underwent automated classification. Then, tweets related to cocaine were analyzed to examine their content, types of Twitter users, usage patterns, health effects, and personal experiences. Geolocation data was also considered to understand regional differences.

**Results:**

A total of 71,844 classifiable tweets were obtained. Among these, 15.95% of users discussed the harm of cocaine consumption to health. Media outlets had the highest number of tweets (35.11%) and the most frequent theme was social/political denunciation (67.88%). Regarding the experience related to consumption, there are more tweets with a negative sentiment. The 9.03% of tweets explicitly mention frequent use of the drug. The continent with the highest number of tweets was America (55.44% of the total).

**Discussion:**

The findings underscore the significance of cocaine as a current social and political issue, with a predominant focus on political and social denunciation in the majority of tweets. Notably, the study reveals a concentration of tweets from the United States and South American countries, reflecting the high prevalence of cocaine-related disorders and overdose cases in these regions. Alarmingly, the study highlights the trivialization of cocaine consumption on Twitter, accompanied by a misleading promotion of its health benefits, emphasizing the urgent need for targeted interventions and antidrug content on social media platforms. Finally, the unexpected advocacy for cocaine by healthcare professionals raises concerns about potential drug abuse within this demographic, warranting further investigation.

## 1 Introduction

Cocaine abuse represents a significant public health concern with relevant medical and socioeconomic consequences worldwide (1, 2). The United Nations Office on Drugs and Crime (UNODC) claims that cocaine is the third type of illicit drug most consumed in the world, just after opiates and cannabis (3). According to their last World Drug Report, approximately 21.5 million people are estimated to have used cocaine in 2020, representing 0.4% of the global population aged between 15 to 64 years old (3). Moreover, the escalating annual trend in cocaine consumption since 2010 underscores the increasing level of concern associated with its use.

Understanding the public´s perception of a drug is essential, as both factors are directly related to its consumption and legislation (4, 5). For instance, previous studies have linked increased cannabis consumption to a perception of low associated risks, influenced partly by varying legislation on medical cannabis use and exposure to related advertising (6–9). Cocaine was first isolated in the middle of the 19^th^ century and gained popularity in the early 1900s (10). However, due to its addictive properties, widespread abuse and related health issues it was banned in the United States in 1914 (11). The public perception of cocaine underwent shifts, notably in the 1970s leading to increased abuse (12). Subsequently, in the 1980s and early 1990s, it became linked to crime, violence, and racial concerns, influencing public policies on its regulation (10). Therefore, analyzing the public perception of drugs, especially cocaine, is crucial for comprehending its current global use/abuse status and the impact of related public policies.

An increasing body of research advocates for the use of social networks as a valuable tool in drug research. They facilitate the understanding and collection of data on social perception, misinformation, and pharmacovigilance (13–15). Twitter is seen as a safe and non-judgmental platform for sharing honest experiences, including sensitive topics like drug use and abuse (16). Previous studies have successfully utilized Twitter as a public health tool to analyze and study drug-related issues (17–19). Artificial intelligence (AI), enables the processing and analysis of vast amounts of data (20). Within AI, Machine Learning (ML) has become a prominent field, focusing on extracting knowledge from data through computational models. A subset of ML known as Deep Learning (DL) employs neural networks inspired by the human brain to process information (21). These neural networks find applications in various domains related to substance use, enabling detection of abuse patterns (22) and related harms (23), also allowing researchers to understand public perceptions and opinions of a drug (5) while exploring potential differences in these points across regions and countries (24). Another essential application is Natural Language Processing (NLP), which extensively utilizes neural networks to analyze text, facilitate conversations, and extract key ideas (25). Most studies conducted on Twitter have focused on cannabis and opioids (5, 18, 26, 27). Currently, some preliminary results related to cocaine use have been obtained from different social media by the use of AI and ML (28, 29) and previous works in Twitter analysis have considered cocaine use in the context of polysubstance use (30, 31). Nevertheless, there is a notable gap in the literature concerning detailed studies collecting information on the use/abuse of cocaine on Twitter through these techniques.

Given the existing gap in detailed studies on cocaine discussions on Twitter, we propose the following hypotheses: First, we hypothesize that through the use of AI and ML, it is possible to find geographical differences in the opinions and concerns expressed about cocaine that reflect unique regional dynamics and social attitudes. Second, we hypothesize that there are distinct considerations related to cocaine based on user profile. Specifically, we anticipate differing opinions among different user groups, such as general or non-identifiable individuals, healthcare professionals, the media, or celebrities language, thereby contributing to a nuanced understanding of the diversity of discourse Finally, we hypothesized that individuals’ personal experiences with cocaine would correlate with their assessment of the risks involved when discussing the substance on Twitter, and that the platform would also collect different frequencies and consumption patterns. This correlation will influence the nature and tone of their contribution to the platform. By addressing these multifaceted aspects, this study aims to provide valuable insights into the complex dynamics of public discourse on cocaine in the digital sphere, providing a comprehensive understanding about the factors that form and differentiate views on this quality.

## 2 Methods

### 2.1 Data collection

This mixed-method, quantitative and qualitative analysis focused on the content of tweets related to cocaine posted on the social media platform Twitter. Our study included tweets that met specific criteria: they had to be public, contain the words “cocaine” or “cocaina,” be published between January 1, 2018, and April 30, 2022, and be in English or Spanish, with a minimum of 10 retweets. These criteria were chosen to ensure a comprehensive and representative sample of social media discussions on the topic. We employed Tweet Binder, a widely used tool in previous research (32–35), to collect the tweets, providing essential information such as retweet and like counts, publication date, tweet context link, user description, and geolocation. The number of retweets and likes served as indicators of user engagement and interest in the tweeted content (36, 37).

### 2.2 Content analysis process

Using the previously mentioned search criteria, we collected 57,192 tweets in Spanish and 54,316 tweets in English. Next, with the remaining tweets, the content was analyzed using a mixed inductive-deductive approach to develop a codebook for classifying the tweets into key thematic categories. A manual classification of a small subset of tweets (n = 100) was conducted by two members of the research team, who later convened to discuss the different categories analyzed. We created a codebook based on our research questions, our previous experience in analyzing tweets, and what we determined to be the most common themes. After discussing discrepancies and reaching a consensus on the codebook, an additional 450 tweets were analyzed. This process also provided a larger sample for training the Machine Learning model. Finally, an automated and computerized classification was performed on the remaining and larger subset of tweets (n = 111,508).

The tweets were classified as classifiable or non-classifiable. A tweet was considered non-classifiable if it was written in a way that made its meaning uncertain, too brief to contain relevant information, if its content was purely political, if the information was not relevant to the objectives of this study, or if it was a joke. In each of the classifiable tweets, the content was analyzed according to the following themes: 1) Tweet topic; 2) Evaluation of the effect; 3) Sentiment regarding consumption; 4) Type of consumption. Finally, the users were classified into four categories: 1) General Twitter users; 2) Media outlets; 3) Public figures; and 4) Healthcare professionals. The classification criteria and examples of tweets are shown in (**Table 1**).

**Table 1:** Category, definitions and examples of classification.

Category	Examples
**Effect assessment** ***(Whether consumption is*** ***perceived as beneficial or a*** ***health risk.)*** **1. Health benefit** **2. Harmful to health**	1. I’m just going to say that cocaine use is destroying a friend and we can’t get him out of there. Stop fucking around. Legal or illegal kills the same.
**Topic** ***1.* Claim (Refers to both** **police/social/** **political complaint/** **claim (for or against))** **2. General information** ***(Refers to when talking*** ***about more scientific issues).*** ***3.* Sale/advertising *(Tobacco*** ***is advertised).*** ***4.* Testimonials *(Regarding*** ***consumption, experience,*** ***more from the opinion of*** ***drug users or families/*** ***friends).*** **5. Trivialization. ** ***(Minimization of the*** ***consequences of*** ***consumption, *** ***stigmatization, humorous*** ***tweets)***	6. The Departmental Anti-drug Brigade arrested a Colombian citizen who was making pink cocaine in an apartment located on Paysandú and Ejido streets in downtown Montevideo. 7. Finally published the analysis I did of 19,000 admissions to mental health hospitalization. There are more and more problems related to cannabis, cocaine and other stimulants and we still do not have a care plan for dual pathology in Andalusia. I want one!
**Personal experience with drugs.** ***(Personal experience with cocaine, whether through acquaintances, friends, family members, or personal use, or related to social events associated with its consumption.)***	1. Impossible to talk to people without culture, mostly high school and called “truckers” enough of “filthy broken” in good Chilean Urgent Railroad PLAN to regulate this plague that HURT the country. BEWARE MANY OF THESE GUYS DRIVE DRUGGED ADDICTED TO COCAINE!!!!
**Consumption type.** ***(Whether it’s about using cocaine frequently, only occasionally, or in binges, not only personal use but also when discussing the consumption of family members or friends.)***	2. Sigrid Alegría confessed in “De tú a tú” about her addiction: “I used cocaine to avoid gaining weight.”
**User type** ***(Refers to the person sharing the tweet.)*** **3. Health professionals.** **4. Undetermined. *(General *** ***population or it is not *** ***possible to identify)*** **5. Media.** **6. Celebrity. (*Any famous *** ***person; singers, actors,*** ***politicians, influencers…).***	7. Cocaine has vasoconstrictive properties which along with other secondary effects lead to ischemia and subsequent perforation of the hard palate (the roof of the mouth). 8. She found that “media reports on crack cocaine frequently referenced African Americans and depicted the drug in conjunction with violent crime. However articles on methamphetamine were more likely to reference poor Whites and associate this drug as a public health problem.” 9. In a single enforcement action #CBP officers at Laredo Port of Entry seize a poly-drug load of black tar heroin brown heroin and cocaine valued at $400K. 10. Cocaine is now legal in Oregon but now straws are illegal. Damn that must be mighty frustrating.

Usernames and personal names were removed.

### 2.3 Machine-learning classifier

The methodology followed in this project has been validated in prior research studies (38, 39). First, a preprocessing of the database should be executed. This preprocessing involves a translation of the non-English tweets to English using Google Translator and a normalization of the tweets by removing special characters, splitting negative contractions, and removing repetitions. Then, we employ a pre-trained network called BERTWEET, trained on 850 million English tweets (40), to classify cocaine-related tweets. Since BERTWEET was not initially designed for the specific classification categories, fine-tuning was performed. Manually classified tweets were randomly divided into an 80% training subset and a 20% testing subset. The training subset was used to fine-tune the network, while the testing subset was used to validate its performance. Additionally, to address some imbalanced categories (where certain options had a higher number of tweets compared to others), text augmentation was performed using the library called textattack (41). Furthermore, emotion analysis was conducted using a pretrained neural network called emotion-english-distilroberta-base (42). This network is capable of detecting six basic emotions according to Ekman’s theory (43) along with neutral sentiment. The emotion analysis was applied to the 71,884 tweets categorized as classifiable.

### 2.4 Statistical analysis

The results were presented in tables or figures, showing the percentage of tweets or the median of likes and retweets in each category. To compare the proportions of tweets between categories, Pearson’s chi-square test was utilized, yielding a p-value indicating statistical significance.

To evaluate the relationships between tweet content, user type, and other tweet characteristics with the number of likes and retweets, linear regression models were employed. The individual beta coefficients were adjusted for the remaining tweet characteristics. Choropleth maps were generated as a visualization tool to depict the global distribution of tweets. Additionally, these maps were used to illustrate the geographic distribution of tweets expressing support for the legislation and exhibiting a sentiment favorable to cocaine.

The statistical analyses were performed using the software packages STATA v16 (StataCorp) and MS Excel.

## 3 Results

### 3.1 Content themes

The study involved analyzing the frequency distribution of tweets across various categories based on tweet characteristics. According to the codebook, a total of 71,844 classifiable tweets were obtained. Among these, 15.95% of users discussed the harm of cocaine consumption to health. Although tweets expressing some health benefits of cocaine receive a higher number of likes, 50% of the tweets have 121.5 likes or more (**Table 2**). Of the total number of users that could be defined, media outlets had the highest number of tweets, with 25,228 tweets (35.11%). The most frequent theme is social or political claims, with 48,768 tweets published, accounting for 67.88% of the total. The least frequent theme is trivialization, but it has a higher number of likes and retweets. Regarding the experience related to consumption, there are more tweets with a negative sentiment compared to a positive sentiment. Approximately 37.07% of the tweets (26,597) display a negative sentiment. Regarding the discourse on cocaine consumption, 9.03% of tweets explicitly mention frequent use of the drug, and they also receive a higher number of likes compared to other subcategories.

**Table 2:** Descriptive characteristics of the tweets are considered classifiable in the content analysis.

	Tweets		Median likes	Median retweet
	n	%	-	-
**Overall**	71,844	100	–	–
*Effect assessment*				
** No mention**	59,282	82.51	65	34
** Health benefit**	1,102	1.53	121.5	29
** Harmful for health**	11,460	15.95	88	32
*User type*				
** Health professionals**	2,030	2,83	87	28
** Undetermined**	37,381	52.03	83	37
** Media**	25,228	35.11	50	30
** Celebrity**	7,205	10.03	85	36
*Topic*				
** Claim**	48,768	67.88	64	35
** General information**	2,230	3.10	60	28
** Sale/advertising**	6,441	8.97	56	36
** Testimonials**	13,316	18.53	98	30
** Trivialization**	1,089	1.52	172	37
*Personal experience with drugs*				
** No mention**	43,257	60.21	60	33
** Positive**	1,990	2.77	174.5	32
** Negative**	26,597	37.02	83	36
*Consumption type*				
** No mention**	63,488	88.31	66	34
** Frequent consumption**	6,491	9.03	101	29
** Occasional/binge consumption**	1,905	2.62	73	27

**Figure 1:**
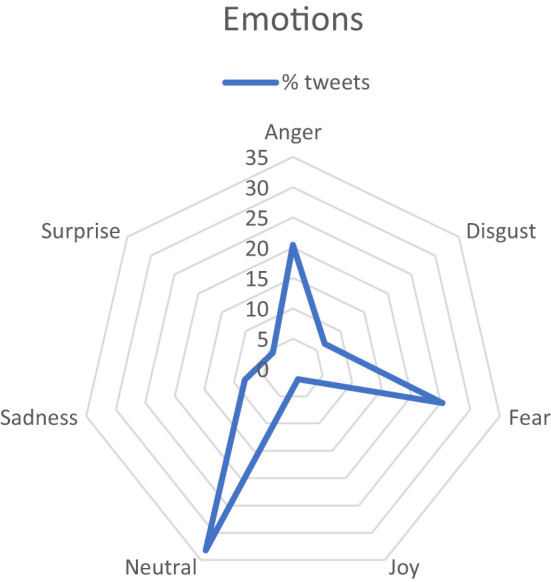
Sentiment analysis (the emotional tone expressed in text). the percentage of tweets associated with each specific emotion.

In terms of emotional expression, the most frequent response from Twitter users is to remain neutral in the majority of their posts, as depicted in **Figure 1**.

**Figure 2:**
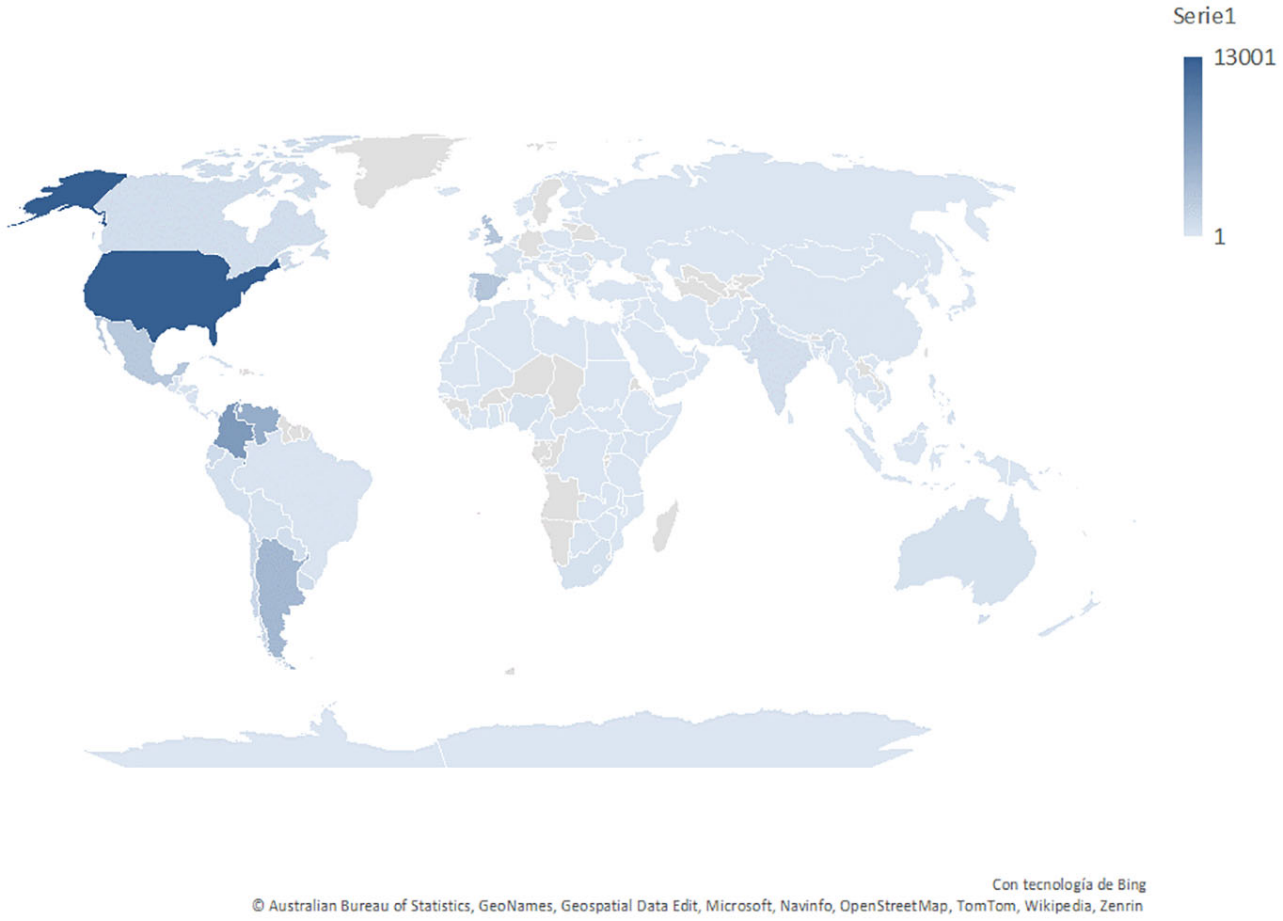
Distribution of the number of tweets worldwide. The area with the highest number of tweets about cocaine is represented with a darker blue color, and the color tone decreases as the number of tweets decreases.

The continent with the highest number of tweets is America, with 39,830 tweets published, accounting for 55.44% of the total. Among the top 5 countries with the highest number of tweets, the first four are from this continent, in descending order: United States, Colombia, Venezuela, and Argentina, representing 41.82% of the total tweets (**Figure 2**).

### 3.2 Geographical analysis

Content analysis by continents reveals that out of the 59,725 geolocated tweets analyzed as shown in (**Table 3**), the most frequent theme across all continents, similar to the overall analysis, is the expression of social/political denunciation, particularly prevalent in America, accounting for 73.05% of the tweets. Regarding the evaluation of the effects, Europe has the highest percentage of tweets discussing the harm caused by cocaine, at 21.63%. Additionally, Asia has the highest proportion of tweets expressing negative sentiment related to consumption, with 41.65% of the tweets falling into this category. Lastly, Africa exhibits the highest content about frequent cocaine use, comprising 13.18% of the tweets.

**Table 3:** Number of tweets by continent and category of the codebook.

	AMERICA	EUROPE	AFRICA	ASIA	OCEANIA
	n (%)	n (%)	n (%)	n (%)	n (%)
*Effect assessment*					
**No mention**	34,233 (85.95)	5,602 (76.51)	986 (77.33)	1,256 (80.10)	630 (86.30)
**Health benefit**	437 (1.10)	136 (1.86)	29 (2.27)	41 (2.61)	12 (1.64)
**Harmful for health**	5,160 (12.96)	1,584 (21.63)	260 (20.39)	271 (17.28)	88 (12.05)
	P<0.001				
*User type*					
**Health professionals**	804 (2.02)	308 (4.21)	44 (3.45)	50(3.19)	24 (3.28)
**Undetermined**	29,984(52.68)	3,802 (42.09)	541 (42.43)	556 (35.46)	311 (42.60)
**Media**	14,711(36.93)	3,139 (42.87)	564 (44.23)	616 (39.29)	305 (41.78)
**Celebrity**	3,311(8.36)	793 (10.83)	126 (9.88)	346 (22.07)	90 (12.32)
	P<0.001				
*Topic*					
**Claim**	29,097 (73.05)	4,323 (59.04)	809 (63.45)	1,003 (63.97)	474 (64.93)
**General information**	945 (2.37)	346 (4.73)	31 (2.43)	40 (2.55)	34 (4.66)
**Sale/advertising**	3,653 (9.17)	483 (6.60)	68 (5.33)	109(6.95)	49 (6.71)
**Testimonials**	5,715 (14.35)	2,060 (28.13)	326 (25.57)	376(23.98)	163 (22.33)
**Trivialization**	420 (1.05)	110(1.5)	41 (3.22)	40 (2.55)	10 (1.37)
	P<0.001				
*Personal experience with drugs*					
**No mention**	26,561 (66.69)	4,093 (55.90)	743 (58.27)	854 (54.46)	404 (55.34)
**Positive**	813 (2.04)	204 (2.79)	70 (5.49)	61 (3.89)	14 (1.94)
**Negative**	12,456 (31.27)	3,025 (41.31)	462 (36.24)	653 (41.65)	312 (42.74)
	P<0.001				
*Consumption type*					
**No mention**	36,139 (90.73)	5,985 (81.74)	1,061 (83.22)	1,397 (89.09)	641 (87.81)
**Frequent consumption**	2,841 (7.13)	929 (12.69)	168 (13.18)	150 (9.57)	72 (9.86)
**Occasional/binge consumption**	850 (2.13)	408 (5.57)	46 (3.61)	21 (1.34)	17 (2.33)
	P<0.001				

### 3.3 User type

If we examine each item concerning types of Twitter users (**Table 4**), it is observed that, about the assessment of cocaine’s effects, the majority of users, excluding healthcare professionals, refrain from mentioning it in their tweet content. Nonetheless, healthcare professionals indicate it as a detriment to health in 82.07% of instances. Additionally, healthcare professionals exhibit the highest percentage (77.04%) of expressing negative experiences related to consumption, followed by public figures, where this aspect appears in 66.74% of the tweets. Finally, regarding the type of consumption, a notably high percentage (81.72%) of healthcare professionals share their perspective on frequent cocaine use.

**Table 4:** Number of tweets by user type and category of the codebook.

	User Type			
	Health Professional	Undetermined	Media	Celebrity
	n (%)	n (%)	n (%)	n (%)
*Effect assessment*				
**No mention**	115 (5.67)	30,418 (81.37)	22,777 (90.28)	5,972 (82.89)
**Health benefit**	249 (12.27)	674 (1.80)	97 (0.38)	82 (1.14)
**Harmful for health**	1,666 (82.07)	6,289 (16.82)	2,354 (9.33)	1,151 (15.98)
	P<0.001			
*Personal experience with drugs*				
**No mention**	242 (11.92)	19,769 (52.89)	21,290 (84.39)	1,956 (27.15)
**Positive**	224 (11.03)	1,414 (3.78)	56 (0.22)	296 (4.11)
**Negative**	1,564 (77.04)	16,198 (43.33)	3,882 (15.39)	4,953 (68.74)
	P=<.001			
*Consumption type*				
**No mention**	345 (17.00)	33,126 (88.62)	23,030 (91.29)	6,947 (96.42)
**Frequent consumption**	1,659 (81.72)	3,440 (9.20)	1,150 (4.56)	242 (3.36)
**Occasional/binge consumption**	26 (1.28)	815 (2.18)	1,048 (4.15)	16 (0.22)
	P<0.001			

### 3.4 The assessment of cocaine’s effects and individual experience, related to the type of consumption

If we relate the evaluation of the effect by Twitter users with those who talk about consumption, it has been observed that 64.34% of the tweets that mention health benefits also mention frequent consumption, nearly double the percentage of those who mention harm to health and frequent consumption, which is 39.73% (**Table 4**).

Regarding individual experiences with the substance, it has been found that almost half (48.14%) of the tweets that speak positively also mention frequent consumption. However, only 17.01% of those who mention harm relate it to frequent consumption (**Table 5**).

**Table 5:** Number of tweets by consumption type and category of the codebook.

Consumption type			
	No mention	Frequent consumption	Occasional/binge consumption
	n (%)	n (%)	n (%)
*Effect assessment*			
**No mention**	57,427 (96.87)	1,229 (2.07)	626 (1.06)
**Health benefit**	392 (35.57)	709 (64.34)	1 (0.09)
**Harmful for health**	5,626 (49.12)	4,553 (39.73)	1,278 (11.15)
	P<0.001		
*Personal experience with drugs*			
**No mention**	41,662 (96.31)	1,010 (2.33)	585 (1.35)
**Positive**	1,025 (51.51)	958 (48.14)	7 (0.35)
**Negative**	20,761 (78.05)	4,526 (17.01)	1,313 (4.94)
	P<0.001		

## 4 Discussion

In the present work, we have collected and classified 71,844 tweets discussing cocaine according to the content of the message, geolocation, type of user, and consumption frequency reported. The results obtained in this article go hand in hand with previous results reported in the Twittersphere in which this type of detail has been studied in other drugs such as opioids or cannabis (30, 44, 45); however, as far as we know this article is the first to deeply explore this type of data about cocaine on this platform.

The majority of analyzed tweets (67.88%) focused on political and social denunciation. Media sources accounted for 35.11% of the tweets, with 55.44% originating from American users who predominantly expressed political and social denunciation (73.05%). These findings highlight evidence cocaine consumption is a significant current social and political issue, particularly in the United States and South American countries. The United States has experienced the highest number of cocaine-related disorders and overdose mortality cases globally (46–48). Recent data from the Centers for Disease Control and prevention (CDC) showed a 54% increase in cocaine-involved deaths, rising from 15,883 in 2019 to 24,486 deaths in 2021 (47). Given these statistics, it is understandable that many tweets from the United States focus on denouncing cocaine abuse from a political and social perspective, emphasizing the need for inclusive public policy reforms (49). In the case of South American countries, a broad number of tweets were identified from Colombia, Venezuela, and Argentina. Colombia in particular has a long history of cocaine trade and continues to be involved in its production and cultivation (10). Twitter and scientific articles discuss the complex sociopolitical context of cocaine crops in this country, analyzing the problem comprehensively (50, 51).

Tweets from Europe and Africa primarily focused on the detrimental health effects of cocaine and the frequent consumption of this drug. In the European Union, 14.4 million people have consumed cocaine at least once in their lives, accounting for 5% of the population (52). Among adults aged 15 to 64, 3.5 million reported cocaine use in the last year, with 2.2 million between the ages of 15 and 34. Cocaine ranked as the second most problematic drug for first-time treatment seekers and the second most commonly reported substance for acute toxicity by Euro-DEN Plus hospitals in 2020 (52). In the same manner, various studies conducted in different European countries have found an increase in cocaine consumption and cocaine-related deaths, also highlighting the multiple health complications related such as psychiatric and psychotic disorders, neurological maladies and cardiovascular diseases (53–55). Thus, our results seem to support that Twitter is seen as a valuable tool to raise awareness about the real problem of cocaine in Europe and its overall negative effects on health. On the other hand, fewer studies are available in the literature regarding cocaine use in Africa. However, different platforms like the Africa Organized Crime Index (56) have evidenced the problem of cocaine trade and abuse in some countries like Guinea-Bissau, Cabo Verde or Guinea, as well as in South Africa or the sub-Saharan countries (57, 58). According to the literature, despite Africa being neither a major producer nor a major consumer of cocaine, the evidence of cocaine’s destabilizing impact has been considered an emerging problem for the last decade (59). The sheer value of the cocaine trade in this region from South American and Caribbean countries poses not only security threats, but also risks distorting the region’s economy, investment flows, development and democracy. Therefore, Twitter can be used as a platform to denounce the habitual consumption of cocaine in this region and the detrimental health effects derived in this region. However, additional efforts in this platform are warranted, particularly in light of our results.

Despite the trivialization of cocaine consumption being the less discussed topic on Twitter, it accumulated almost double the interactions with other Twitter users (172 likes and 37 retweets versus 64 likes and 35 retweets), as well as those reporting positive versus negative effects. In addition, when considering the type of cocaine consumption on Twitter, frequent consumption was more common than occasional use (9.03% versus 2.62%), also receiving more interactions. Previous research has indicated that drugs are often discussed positively on social media platforms like Twitter, and the lack of antidrug content may contribute to the normalization and justification of drug use, highlighting the importance of addressing this issue (60). Furthermore, the dissemination of trivialization may contribute to an increase in hospitalizations due to cocaine consumption, even in the pediatric population (61). In agreement with previous works (62, 63), our results support the notion that social media like Twitter can serve as valuable resources for understanding drug patterns, prevailing attitudes, monitoring and intervening in drug abuse and addiction problems.

We found a small proportion of tweets promoting the supposed health benefits of cocaine use, which received significant engagement. This is an important issue to address, as there are no safe ways to consume cocaine. Misconceptions regarding the health benefits of cocaine may stem from historical events and practices, such as its traditional use in South America for over 5,000 years as a stimulant in the form of teas or by chewing the leaves of the *Erythroxylon coca* plant (64). Additionally, influential figures like Sigmund Freud, as well as the incorporation of cocaine in beverages like Coca-Cola and coca wine during the late 19th and early 20th centuries, contributed to its popularity (11). As previously mentioned, despite being banned in the USA in 1914, during the 1970s, cocaine regained a positive image, fueled by perceptions of glamour and media influence. Even the Ford White House in 1975 released a white paper stating that cocaine was not physically addictive and generally did not have serious consequences (12). Conversely, cocaine use leads to a wide range of harmful effects including tachycardia, hypertension, acute coronary syndrome, stroke, and even death (65). Mixing cocaine with substances like sugar, talc, and cornstarch exacerbates these adverse effects (66). Factors such as high drug purity, frequent or binge consumption and polydrug use (particularly with alcohol and fentanyl/heroin) contribute to toxicity and overdose risks (67–69). Previous Twitter analyses have shown that polysubstance use involving cocaine and other drugs is a common topic in discussions about overdose and drug-related concerns (18, 30, 31, 70). Although our study did not focus on polydrug use, it is important to consider these findings, as the low perception of risks associated with cocaine use obtained in our study may even be more concerning in such contexts. Furthermore, long-term consumption of cocaine is associated with significant brain changes in the dopaminergic reward system, resulting in addiction, persistent cravings and a high risk of relapse, even with treatment (71). Cocaine use disorder (CUD) represents a serious global health concern, and while psychosocial and pharmacological interventions can assist in the medical management of this condition, the efficacy is limited and ineffective for most patients (72). Moreover, despite some specific clinical cases in the 20^th^ century, the risks of cocaine use outweigh any potential benefits, and there are safer alternatives for various purposes attributed to this substance (10).

Therefore, it is crucial to address and intervene in the content on Twitter that trivializes or supports the alleged health benefits of cocaine use.

Intriguingly, our study shows that healthcare professionals on Twitter were among the strongest advocates for the health benefits, frequent use and positive experiences related to cocaine (12.27%, 81.72% and 11.03%, respectively). This could be relevant considering previous studies that have identified drug abuse among healthcare professionals as a concern (73), especially when considering certain risk factors such as certain medical specialties, psychopathological or social factors, positive attitudes toward drugs, unhealthy lifestyle habits and so on (73). Although we could not explore all contributing factors, further investigation is needed to understand the relationship between drug abuse and healthcare professionals on social media platforms like Twitter, as our findings imply that they may use it to share personal experiences and concerns related to drug use and abuse.

Finally, we also observed a notable proportion of tweets (8.97%) showing sale/advertising content. This is not a novel issue as previous works have also identified social media like Twitter as a conduit for the sale and supply of illicit drugs like opioids (74, 75). We encourage the regulation of this type of illegal cocaine sale, proposing the inclusion and use of possible programs implicated in the detection, classification and reporting of illicit online sale tweets, as promoted in previous works (76).

## 5 Limitations

This research has some notable limitations. Firstly, Twitter users’ social, economic, and demographic attributes do not accurately mirror the entire society. Second, just like practically all qualitative investigations, the construction of the codebook and the analysis of the tweets involve certain subjectivity. Third, there is a chance that we overlooked tweets that made reference to cocaine but did so in slang or contractions like “coke”, “C”, “snow”, “flake” and “blow”. Similarly, it is also possible that bots or fake accounts have to some extent affected our data. Finally, the inclusion of tweets with 10 or more retweets could also be a limitation of the study, as it might have overlooked relevant tweets for this article.

## Data availability statement

The raw data supporting the conclusions of this article will be made available by the authors, without undue reservation.

## Ethics statement

This study was approved by the Research Ethics Committee of Universidad de Alcalá and is compliant with the ethical principles from the World Medical Association Declaration of Helsinki (7th revision, 2013).

## Author contributions

CCT: Data curation, Investigation, Methodology, Validation, Writing – original draft, Writing – review & editing. OF-M: Conceptualization, Validation, Writing – original draft, Writing – review & editing. CD-V: Formal analysis, Investigation, Methodology, Writing – review & editing. FL-A: Formal analysis, Investigation, Software, Writing – review & editing. MO: Conceptualization, Supervision, Validation, Writing – original draft, Writing – review & editing. CG-M: Supervision, Validation, Writing – original draft, Writing – review & editing. FM: Conceptualization, Resources, Visualization, Writing – review & editing. MA-M: Conceptualization, Funding acquisition, Resources, Supervision, Writing – review & editing. JQ: Methodology, Supervision, Visualization, Writing – review & editing. MAA-M: Conceptualization, Investigation, Methodology, Project administration, Resources, Visualization, Writing – original draft, Writing – review & editing.
